# Implementing a food first strategy can transform preventive healthcare

**DOI:** 10.1038/s41538-024-00297-4

**Published:** 2024-08-27

**Authors:** Zhaoshuo Yu, Lijing Ke, Ting Lu, Li Li, Huaiyu Gu, Pingfan Rao

**Affiliations:** 1 National Nutrition Surveillance Centre, University College Dublin, Belfield, Dublin 4 Ireland; 2 School of Agriculture and Food Science, University College Dublin, Belfield, Dublin 4 Ireland; 3 School of Food Science and Nutrition, University of Leeds, Leeds, LS2 9JT UK; 4 Hangzhou Normal University, 311121 Hangzhou, China; 5 Institute of Basic Medical Sciences, Chinese Academy of Medical Sciences; Department of Human Anatomy, Histology and Embryology, School of Basic Medicine, Peking Union Medical College, 100005 Beijing, China; 6 International Union of Food Science and Technology (IUFoST), Guelph, ON Canada

**Keywords:** Biotechnology, Health care

## Abstract

The Food-First Strategy advocates seeking a nutritional solution for the prevention and treatment of disease before resorting to supplements or therapeutic agents. Advances in knowledge of nutrition at the cellular level are providing information on how micronutrients are incorporated into cells and how they exert their actions. Micronutrients, in the form of naturally occurring nanoparticles, are more bioavailable and also act as antioxidants to tackle inflammation and promote cellular regeneration and repair. They are the new “superheroes of nutrition” and an understanding of their metabolic impact can explain and support associated health claims.

## Introduction

With the exponential increase in the costs associated with illness across all jurisdictions globally, it is imperative to re-evaluate the approach to disease prevention and management. In this discourse, we advocate for a paradigm shift—a departure from the prevalent therapeutic-centric model towards a Food-First Strategy. China, with its rich tradition of linking food and health, offers a compelling blueprint for this transformative approach^1,2^. The Food-First Strategy prioritises dietary solutions, before resorting to supplements and pharmaceutical agents, for the prevention and management of disease.

Ancient civilizations, from the Ayurvedic traditions of India to the Hippocratic school of ancient Greece, recognized the therapeutic potential of food. Hippocrates, often regarded as the father of Western medicine, famously proclaimed, “Let food be thy medicine and medicine be thy food^3^”. Across diverse cultures, indigenous healing traditions have revered specific foods for their medicinal virtues, harnessing herbs, spices, and culinary techniques to alleviate ailments and promote vitality^4–6^. From the pharmacopoeias of traditional Chinese medicine^7^ to the indigenous botanical knowledge of Amazonian shamans^8^, the historical tapestry of food as medicine bears testament to humanity’s innate wisdom in leveraging nature’s pharmacy for holistic well-being.

Historically, Chinese culture has recognized the intrinsic relationship between food and well-being, a notion deeply ingrained in its culinary practices^2^. Contrastingly, in much of the Western world, food has been relegated to a mere commodity, overshadowing its potential as a potent preventive and therapeutic tool. In most major cities globally, the proliferation of pharmacies, often open 24 h per day seven days per week, has become emblematic of a societal shift towards a pharmaceutical-centric approach to health. These establishments offer a convenient avenue for individuals seeking their micronutrients in a supplement format, perpetuating the perception that health can be encapsulated in a pill^9^. Furthermore, increasingly in many jurisdictions visits to medical professionals are often perceived as incomplete without a prescription for a pharmaceutical intervention. This situation subtly redefines healthcare providers as “sickness professionals,” emphasizing their role in managing illness rather than fostering holistic well-being^10^. As a consequence, the emphasis on therapeutic solutions is eclipsing the potential of preventive measures, perpetuating a cycle of dependency on pharmaceutical interventions. By contrast, the ‘Food-First Strategy” espoused in traditional Chinese culinary practices offers a departure from this reductionist approach, championing the intrinsic link between food and health as a cornerstone of preventive healthcare^11^.

Traditional beliefs on the relationship between food and health lack supporting scientific evidence and are treated with scepticism by the medical profession and often dismissed as “no better than oldwives tales. However, it is interesting that consumers in many jurisdictions are receptive to the spurious claims associated with many supplements. Whereas varieties of supplements come and go, many traditional beliefs have stood the test of time.

Table 1 lists various foods along with their traditional beliefs and corresponding scientific evidence. However, much of this evidence is either insufficiently disseminated, often published in journals that sceptics may not read, or it lacks the robustness needed to meet rigorous scientific scrutiny.

**Table 1:** The traditional belief and scientific validation associated with some foods

Food	Traditional belief	Area	Nutrients /Bioactives	Scientific validation	Reference
Green tea	Anti-aging and improved mental clarity	Asia	Catechin	Tea consumption was associated with reduced biological ageing, especially for consistent tea drinkers with moderate consumption, based on a cohort study of nearly 14,000 participants.	Xiang et al.^52^
Turmeric	Manage inflammatory conditions and improve Osteoarthritis	India	Curcumin	After an intake of 1000 mg / day for 4-16 weeks results showed improvements in all clinical symptoms of Osteoarthritis (OA) through a meta-analysis of 724 OA case studies.	Mathieu et al.^53^
Bone broth	Improve immunity and improve digestive functions	East Asia and Middle East	Collagen, Glycosamin-oglycan	Oral Hyaluronic acid reduced the incidence and severity of disease in a mouse model of necrotizing enterocolitis.	Gunasekaran et al.^54^
Bone broth	Improve Osteoarthritis	East Asia and Middle East	Collagen, Glycosamin-oglycan	Yak bone collagen significantly alleviated the symptoms of osteoporosis in ovariectomized rats in a dose-dependent manner	Ye et al.^55^
Sweet Wormwood herb (Qinghao)	Alleviate malaria symptoms	China	Artemisinin (Qinghaosu)	Based on numerous clinical trials, it is now recommended by the WHO as the first-line treatment for all falciparum malaria in malaria endemic countries.	Tu. 2011^56^, White.^57^, Li et al.^58^

A pivotal aspect of the Food-First Strategy lies in the deeper understanding of the complicated direct interaction of food with the immune system along the whole alimentary tract. While the Western approach often leans towards supplement consumption, the inefficiency of this method^12^ in comparison to nutrient-rich foods is stark. Micronutrients embedded within a food matrix exhibit superior bioavailability and therefore substantial bioactivity, a phenomenon underscored by centuries of culinary wisdom in China^13,14^.

## Example: joint health

Visitors to China often marvel at the agility and vitality displayed by the elderly as they gracefully practice Tai Chi in public parks. Remarkably, the elderly in China exhibit lower rates of osteoarthritis compared to Western nations, with a notable absence of long queues for invasive procedures like hip and knee replacements or surgeries for prolapsed intervertebral discs^15,16^. While some attribute this phenomenon to genetic predispositions, studies reveal a different narrative^17^. Chinese individuals who migrate to the United States and are exposed to a Western lifestyle, soon experience the musculoskeletal ailments prevalent in their Western counterparts^18^. This suggests that factors beyond genetics play a pivotal role in preserving joint health among the Chinese populace.

As one example of the Food-First strategic approach, we investigate the fundamental role of nourishment in supporting joint health within the traditional Chinese cuisine. We propose that specific dishes unique to the domestic Chinese diet may serve as potent sources of nutrients crucial for the maintenance and repair of cartilage, ligaments, and joints. Three commonly domestically consumed dishes, bone soup, bovine trachea, and chicken feet stand out as they, encapsulate a spectrum of components essential for cartilage health. These include proteoglycans such as aggrecan, versican, decorin, and biglycan, as well as glycosaminoglycans like chondroitin glucosamine and hyaluronic acid, alongside the collagen constituents such as proline-, glycine-, and hydroxyproline-rich peptides^19–21^. Given the poor blood supply to cartilage it is more likely that repair and regeneration will occur if the blood is saturated with the essential micronutrients required^22^.

Hyaline Cartilage, predominant in joints and the developing skeleton, exhibits minimal vascularity, relying on synovial fluid within the joint cavity for nutrient exchange^23^. In contrast, fibrocartilage, found in regions requiring greater tensile strength such as intervertebral discs and the pubic symphysis, possesses slightly enhanced vascularity, albeit still lacking direct blood supply^24^. Nutrient exchange occurs primarily through diffusion from adjacent blood vessels in surrounding tissues. We postulate that micronutrients crucial for cartilage repair may penetrate the extracellular matrix through mechanisms involving diffusion or active transport, thereby replenishing, and revitalizing joint tissues. Injuries to tendons, ligaments, and cartilage often disrupt training and can end the careers of amateur and professional sportspeople when both surgical and non-surgical treatments fail. A nutritional approach to enhance the repair and regeneration of these tissues could be a game changer, revolutionizing the health and longevity of all sports participants.

## Natural Food Nanoparticles

**Fig. 1:**
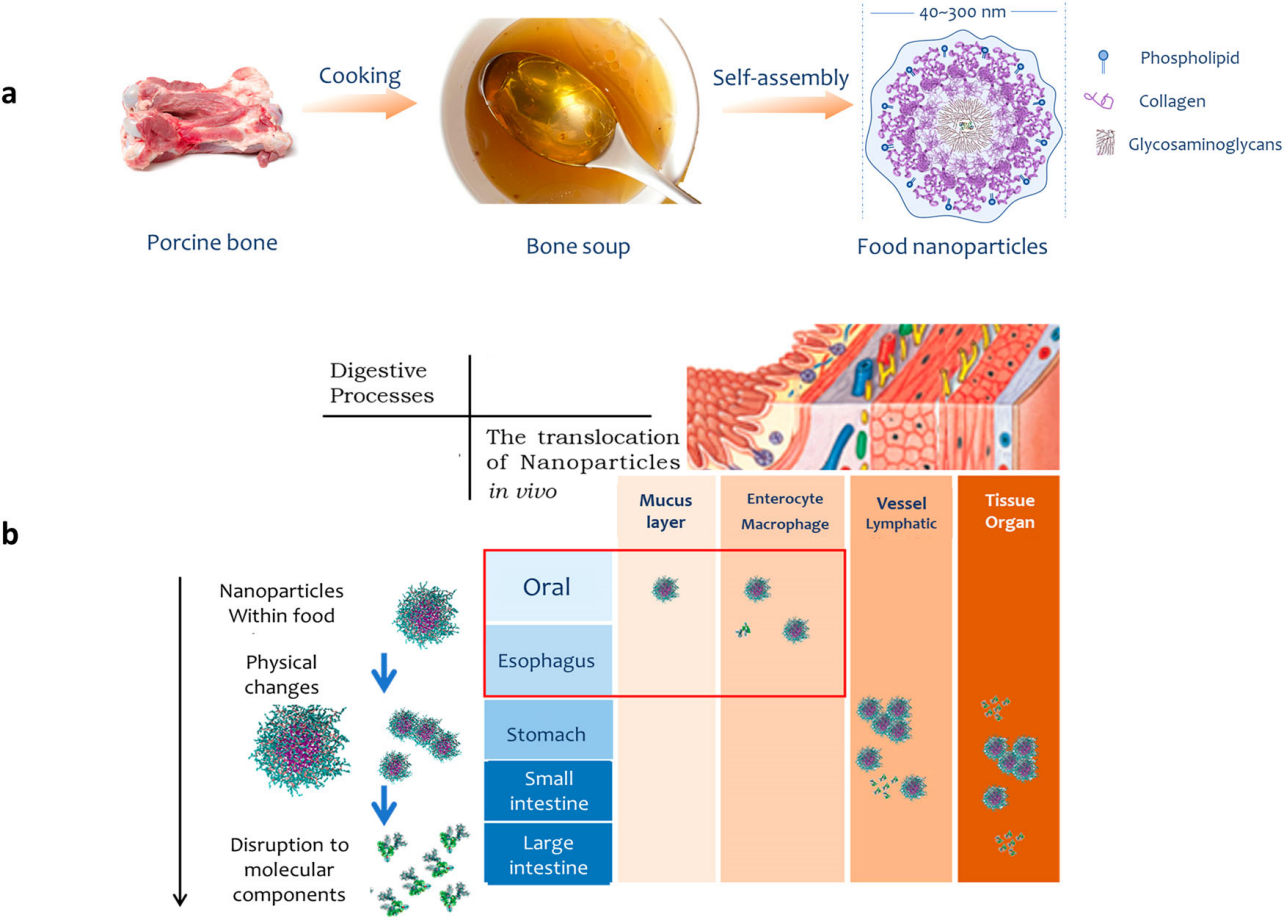
The formation and translocation of natural food nanoparticles entering human body through the gastrointestinal tract (GIT). **a** Cooking of bone soup results in the formation of natural nanoparticles. **b** The digestive processes and translocation of natural food nanoparticles in GIT.

Not only do these dishes provide essential building blocks for cartilage, but they also undergo a culinary alchemy during cooking, yielding natural nanoparticles, minute particles between ten to 1000 nm in size^25,26^. During food processing, the components are released from the original food cells and aggregated into nanoparticles following a series of physical and chemical reactions. For example, cooking of porcine bone soup results in plentiful natural nanoparticles ranging from 40 to 300 nm and these nanoparticles are absorbed along the full length of the gastrointestinal tract (Fig. 1).

Big pharmaceutical companies are increasingly investing in cutting-edge technologies, in attempting to develop expensive nanoparticles for drug delivery systems. These engineered nanoparticles hold promise for targeted drug delivery and enhanced therapeutic efficacy^27^. However, the naturally occurring nanoparticles found in various foods from both plant and animal sources—a marvel of nature’s design—often go unnoticed^28^. Unlike their synthetic counterparts, these natural nanoparticles are endowed with unique properties that facilitate efficient nutrient delivery and biological activity within the body, while being totally biocompatible and biodegradable^29^. Yet, their potential remains largely untapped and underappreciated. It is a testament to the prevailing bias towards synthetic solutions that the invaluable contributions of naturally occurring nanoparticles in food are overlooked. As we champion the Food First strategy, it is imperative to recognize and harness the inherent benefits of these natural nanoparticles, offering a cost-effective and sustainable alternative to pharmaceutical interventions.

Natural nanoparticles exhibit unique physicochemical properties due to their small size and high surface area-to-volume ratio which contributes to both their nutritional profile and functionality^30^. Detecting and characterizing natural nanoparticles in food is challenging due to their small size and potential interaction with other food components. Advanced techniques such as electron microscopy, dynamic light scattering, and spectroscopic methods are typically employed for their analysis^31^.

**Fig. 2:**
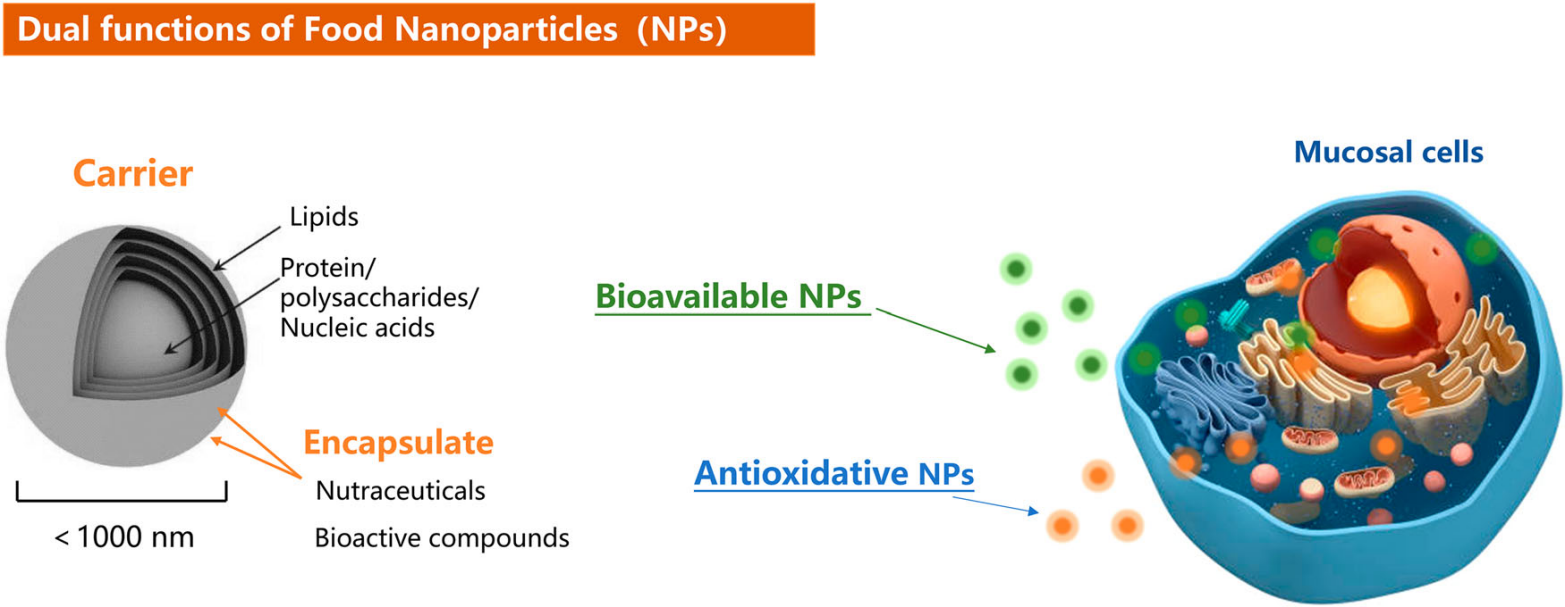
The beneficial effects of natural food nanoparticles on gastrointestinal mucosal cells. Food macromolecules such as polysaccharides, proteins, lipids, and nucleic acids gradually form the multi-layered framework of nano-carriers, encapsulating nutrients and bioactive compounds. This process results in the production of nanoparticles that enhance bioavailability and possess bioactive properties, such as antioxidant activity.

These minute colloidal particles are truly the superheroes of the nutrition world and serve a dual purpose, as shown in Fig. 2^32^. Firstly, facilitating the release of essential micronutrients from food matrices, improving their bioavailability by enhancing their absorption and utilization within the body and their miniature size confers a distinct advantage, ensuring efficient delivery to target tissues and organs—an attribute lacking in conventional supplement formulations^33^.

Secondly, these food nanoparticles function as potent antioxidants, counteracting free radicals and mitigating oxidative stress—a hallmark of numerous chronic diseases^34,35^. By bolstering cellular repair mechanisms, suppressing inflammation and supporting enzymatic reactions, they foster an environment conducive to DNA repair, cell proliferation, and tissue regeneration^36,37^. The dual functions of naturally occurring nanoparticles in food provides an advantage over nutritional supplements, as their small size enhances bioavailability, while their antioxidant effect reduces inflammation and facilitates cell repair (Fig. 2).

## Health claims

In many jurisdictions, stringent regulations govern health claims associated with food products, aimed at safeguarding consumer interests, and promoting evidence-based dietary choices. These regulations necessitate robust scientific substantiation for any health-related assertions made by food manufacturers, ensuring transparency and accountability in the marketplace^38^. However, juxtaposed against these regulatory frameworks are the deeply entrenched traditional beliefs surrounding the health benefits of certain foods, passed down through generations in diverse societies. These beliefs often lack empirical validation but resonate deeply within cultural narratives, shaping dietary preferences and consumption patterns. For example, in China, in addition to the benefits for joint health^39^, traditional beliefs hold that bone broth is good for digestive function^40^, strengthening immunity^41,42^, reducing fatigue^43^ and improving satiety^44^.

Reconciling traditional wisdom with contemporary scientific standards has posed a formidable challenge for food producers seeking to make health claims on traditional products. The absence of rigorous scientific data to support these traditional claims, leaves food businesses exposed to the allegation that they are disseminating potentially misleading information to consumers. While anecdotal claims rooted in tradition abound, the current practice, prior to regulatory acceptance, is to have these assertions substantiated with empirical evidence derived from rigorous and costly dietary intervention studies and clinical trials. However, in the pursuit of validating health claims associated with traditional foods within the Food-First strategy, the emergence of innovative technologies and scientific methodologies offers a promising avenue for elucidating their physiological mechanisms of action. As shown in Table 2, recent advancements in fields such as cell metabolism, nanoscience, and immunology, coupled with our deepening understanding of cellular repair and antioxidant mechanisms, provide unprecedented opportunities to unravel the intricacies of nutritional bioactivity^45,46^. For instance, elucidating the role of immune cells in nutrient absorption sheds light on the dynamic interplay between dietary constituents and immune responses within the gut microenvironment^35,47^ The nanoparticles isolated from porcine bone soup have been found to be engulfed by macrophages and possess antioxidant and anti-inflammatory activities upon intestinal cells^26^, tissues, and microbiota^35^. Furthermore, information from the pharmaceutical sector is demonstrating nanoscale delivery systems allowing for precise targeting of bioactive compounds to cellular sites of action, optimizing their therapeutic potential^48^. The naturally occurring nanoparticles in food are likely to operate in the same way and perhaps more efficiently^36^. By elucidating the molecular pathways through which nutrients exert their beneficial effects, modern food scientists, physiologists, and biochemists are poised to elevate traditional health claims from the realm of anecdote to evidence-based practice. Consequently, standard assessments of health claims by regulatory authorities should evolve to incorporate insights gleaned from these innovative technologies, ensuring a comprehensive and nuanced understanding of the health-promoting properties inherent in traditional foods. In doing so, we bridge the gap between tradition and science, fostering a paradigm wherein ancestral wisdom finds resonance in contemporary evidence-based practice, ultimately advancing public health outcomes on a global scale.

**Table 2:** The sources and functions as alternative pharmaceutical intervention of food nanoparticles

NPs Source	Food	Type of study	Effects	Reference
Mammal	Porcine bone soup	Animal model	NPs isolated from porcine bone soup ameliorated dextran sulfate sodium-induced colitis and regulated gut microbiota in mice	Wang et al.^35^
Mammal	Porcine bone soup	Animal model, Cell model	NPs derived from porcine bone soup reduced oxidative stress-induced intestinal barrier injury in Caco-2 cell monolayer model.	Gao et al.^59^
Mammal	Milk	Animal model	Milk-derived nanoparticles protected intestinal barrier integrity in the gut-liver axis	Tong et al.^60^
Poultry	Duck soup	Cell model	NPs from duck soup exhibited antioxidant effect on macrophages and enterocytes	Xu et al.^37^
Aquatic animal	Clam soup	Animal model	NPs from freshwater clam soup alleviated non-alcoholic fatty liver disease of *Meriones unguieulataus*	Yu et al.^61^
Aquatic animal	Oyster	Animal model, Cell model	Oyster mantle-derived exosomes alleviate osteoporosis by regulating bone homeostasis	Hu et al.^62^
Plant	Herbal decoction	Cell model	Polysaccharide NPs from Isatis indigotica Fort. Root Decoction exhibited antiviral activity	Gao et al.^63^
Plant	Black tea	Animal model, Cell model	NPs in black tea alleviated DSS-induced ulcerative colitis in BALB/c mice.	Han et al.^64^
Plant	Rice vinegar	Cell model	NPs isolated from fermented rice vinegar reduced the oxidative stress within macrophages	Yu et al.^34^
Plant	Ginseng	Animal model, Cell model	Anti-glioma effect of ginseng-derived nanoparticles by active blood–brain-barrier penetration and tumor microenvironment modulation	Kim et al.^65^
Plant	Pueraria lobata	Animal model, Cell model	Pueraria lobata-derived nanoparticles alleviated osteoporosis by enhancing autophagy	Zhan et al.^66^

The improved bioavailability of micronutrients associated with natural nanoparticles is achieved by both their small size and their relatively large surface area which facilitates interaction with biological surfaces and the more effective cross-membranes transportation.

## Pivotal role of food in health

In the paradigm shift towards a Food First strategy, it becomes evident that conventional notions of healthcare providers must evolve. While doctors and nurses continue to navigate the realm of illness management, it is those dedicated to nutrition, exercise, and mindfulness who embody the essence of true health professionals. As such, the onus falls not only on individuals but also on food businesses to acknowledge their pivotal role in fostering health and well-being. Regrettably, the elderly, among the most vulnerable demographic in society, have been let down by the commodification of food and the proliferation of therapeutic solutions. Food businesses must accept their responsibility and assert their role in the health domain. By prioritizing the production and promotion of nutrient-rich, wholesome foods, they can reclaim their position as genuine contributors to public health, challenging the dominance of the supplements sector and big Pharma and forge a path towards a healthier future for all.

Polypharmacy has reached epidemic proportions among the elderly in Western societies. Considering that all drugs carry potential side effects and given the diminished efficiency of the kidneys and livers of the elderly, as well as their declining cognitive function, the risks of overdosing and experiencing adverse drug reactions are huge^49–51^. Therefore, it is imperative for those promoting health to explore all conceivable nutritional avenues before resorting to expensive mixtures of pharmaceutical agents laden with potential toxic side effects.

The implications of embracing a Food First Strategy extend far beyond individual health outcomes. By prioritizing nutrient-rich diets over pharmaceutical interventions, we pave the way for a more sustainable and equitable healthcare system. Reduced reliance on synthetic supplements, and therapeutic agents, alleviates strain on healthcare infrastructure, democratizing access to preventive measures across socio-economic strata.

Moreover, this shift heralds a renaissance in culinary traditions, fostering a renewed appreciation for indigenous ingredients and culinary techniques. By marrying ancient wisdom with modern science, we unlock the full potential of food as medicine—a testament to the enduring synergy between human health and culinary heritage.

The Food First strategy represents a watershed moment in the evolution of preventive healthcare—an ethos rooted in holistic wellness and culinary ingenuity. As we navigate the complexities of modernity, let us heed the lessons of the past and embrace the transformative power of food in safeguarding our collective well-being.

The health of a nation’s citizens is fundamental to the sustainable development of a country’s economy. It is imperative to recognize the pivotal role of food and health in this equation. Governments must recalibrate their priorities to position public health at the forefront, acknowledging not only the burden of diet related diseases stemming from unhealthy consumption patterns but also emphasizing the transformative potential of proper nutrition in disease prevention and treatment. Exploiting the Food First Strategy will chart a course towards a healthier, more resilient future for generations to come.
